# Aspirin inhibited the metastasis of colon cancer cells by inhibiting the expression of toll-like receptor 4

**DOI:** 10.1186/s13578-017-0198-7

**Published:** 2018-01-04

**Authors:** Jun Ying, Hai-yang Zhou, Peng Liu, Qing You, Fei Kuang, Yi-nan Shen, Zhi-qian Hu

**Affiliations:** 1 Department of Surgery, Changzheng Hospital, The Second Military Medical University, 415 Fengyang Road, Shanghai, 200003 China; 2 Department of Surgery, Changhai Hospital, The Second Military Medical University, Shanghai, China; 3 Department of Gastrointestinal Surgery, Shanghai Shuguang Hospital, Shanghai University of Traditional Chinese Medicine, Shanghai, China; 4 Emergency General Surgery, Changhai Hospital, The Second Military Medical University, Shanghai, China

**Keywords:** Aspirin, Lipopolysaccharide, Colon cancer, Epithelial-mesenchymal transition, Toll-like receptor 4

## Abstract

**Background:**

The metastasis of colorectal cancer frequently tends to liver, which is one of the three leading causes of cancer-related deaths worldwide. Growing evidence showed that aspirin could effectively inhibit liver metastasis of colorectal cancer. However, the potential mechanism has not been fully understood.

**Methods:**

Mouse splenic vein metastasis assay was used to examine the metastatic ability of colon cancer cells in vivo. And wound healing and transwell assay were applied to detect the metastasis potential of C26 and HCT116 colon cancer cell lines in vitro. RT-PCR and western blotting were used to explore Toll-like receptor 4 (TLR4) expression in colon cancer cell lines. The functions of TLR4 in the migration of the colon cancer cell line were analyzed by infecting cells with lentivirus containing TLR4 siRNA.

**Results:**

We demonstrated that lipopolysaccharides (LPS) could enhance the metastasis potential of C26 and HCT116 colon cancer cell lines. However, aspirin effectively decreased the metastasis capacity of colon cancer cells in vitro and in vivo. We found that the enhancement of LPS on the migration of colon cancer cells by inducing epithelial-mesenchymal transition (EMT) phenotype demonstrated a TLR4-dependent manner. Aspirin treatment lead to the downregulation of TLR4 on C26 cells which resulted in the decrease of C26 cells migration and EMT phenotype that induced by LPS. Additionally, the inhibitory effect from aspirin on the expression of TLR4 on C26 cells leads to the downregulation of NF-κB.

**Conclusion:**

The results of our study indicate that LPS origin from intestinal flora may promote the metastasis of colon cancer to liver and aspirin may inhibit the metastasis of colon cancer by inhibiting the expression of TLR4.

## Background

Colorectal cancer (CRC) is one of the most common malignancies worldwide, which has become a major cause of death in developed countries. Each year, there are more than 400,000 of new cases and lead to 212,000 death [1]. The liver is the most common metastasis organ of CRC. About 10–25% CRC patients in the diagnosis are found liver metastasis and 20–25% of the patients occur liver metastasis after surgery treatment [2]. Liver metastasis has become a bottleneck for the treatment result and prognosis of CRC [3].

Aspirin, also known as acetylsalicylic acid (acetylsalicylic acid, ASA), is daily used as antipyretic analgesics. Aspirin use is associated with the inhibitory effect of breast, esophageal, lung, stomach, ovarian and colorectal cancer, and aspirin is both a chemopreventive and chemotherapeutic agent for breast and colon cancer [4–8]. It has been reported that aspirin not only can reduce the occurrence of CRC but also reduces the risk of metastasis in CRC [9, 10]. A recent report on the chemopreventive effects of aspirin showed that the incidence of colon cancer in was significantly decreased [7]. However, aspirin’s mechanism of action as an anticancer agent remains unknown.

Lipopolysaccharide (LPS) is a component of cytoderm from gram-negative bacteria, which is released during lysis of bacteria. The level of LPS always increases in the enteric cavity and portal venous blood of the patients with CRC because of the intestinal obstruction, increased gut permeability, or bacterial overgrowth. Besides that, LPS also play an important role in occurrence, development and metastasis of tumors [11–13]. In addition, LPS could effectively increase liver metastasis of human CRC cells [14, 15].

However, the intervention effect of aspirin on the CRC metastasis induced by LPS has not been fully documented. In this study, we observed the role of LPS on the metastasis potential of two colon cancer cell lines, C26 and HCT116. Furthermore, the effect of aspirin on metastasis capacity of colon cancer cells was examined. Besides that, the potential mechanism of aspirin on the colon cancer cells metastasis induced by LPS has been explored.

## Methods

### Reagents

LPS derived from Escherichia coli strain 055:B5, Trizol, Lipofectamine 2000 and BAY11-7028, the inhibitor of nuclear factor kappa B (NF-κB) and aspirin were purchased from Sigma (St. Louis, MO, USA).

### Cells and animals

The BALB/c mice colon cancer cell line C26 and human colon cancer cell line HCT116 were used in this study, both of them were maintained in RPMI 1640 culture medium (GIBCO, Invitrogen, Carlsbad, CA, USA) supplemented with 10% fetal bovine serum (FBS; GIBCO, Invitrogen), 100 units/ml penicillin and 100 mg/ml streptomycin in a humidified incubator under 95% air and 5% CO_2_ at 37 °C.

Male Balb/c mice, 6–8 weeks old, were purchased from Shanghai Experimental Animal Center of the Chinese Academy of Sciences, Shanghai, China. Mice in this study were housed in pathogen-free conditions. All animals’ experimental procedures were performed in accordance with the institutional ethical guidelines from the Animal Ethics Committee of the Second Military Medical University, Shanghai, China.

### Mouse splenic vein metastasis assay

C26 cells (5 × 10^5^/mouse) were injected in 8-week-old Balb/c mice via the splenic vein. The mice were sacrificed after 4 weeks. Surface liver metastasis was measured.

### Wound healing and transwell assay

The methods for wound healing and the Transwell assay have been described [16, 17]. In wound healing assays, migration of the cells was assessed by measuring the movement of cells into a scarped. The speed of wound closure was monitored after 24 h by measuring the ratio of the size of the wound at 0 h.

### Real-time PCR

Total RNA was isolated using TRIZOL (Invitrogen, Carls-bad, CA, USA) and cDNA synthesis was performed using the PrimeScript RT reagent Kit (Takara, Kyoto, Japan) according to the manufacturer’s instructions. The mRNA expression of EMT markers (E-cadherin and Vimentin) was quantified by real-time quantitative PCR. Quantitative PCR was performed using SYBR Green PCR Kit (Applied BI) according to the manufacturer’s instructions.

### Western blot

The treated cells were washed with PBS and lysed by RIPA and PMSF at a ratio of 100:1 to obtain the total protein for Western blot. Equal amount of proteins was separated by SDS-PAGE and transferred to polyvinylidene fluoride membrane. After transferring, the polyvinylidene fluoride membrane was blocked in 5% fat-free milk/1 × TBS/0.1% Tween-20 for 1 h at room temperature and then incubated with primary antibodies overnight at 4 °C. And then, the membrane was washed with 1 × TBS/0.1% Tween-20 before incubated with the secondary antibody for 1 h at room temperature. Immunoblots were performed by using the BeyoECL Plus substrate system (Beyotime), followed by washing with 1 × TBS/0.1% Tween-20.

### Cell transfection

Small interfering RNA (TLR4 siRNA) was added according to the manufacturer’s instructions. The sequences specific for TLR4: sense 5′-GATCCCGTTGAAACTGCAATCAAGAGTGTTGATATCCGCACTCTTGATTGCAGTTTCAATTTTTTCCAAA-3′; anti-sense: 5′-AGCTTTTGGAAAAAATTGAAACT GCAATCAAGAGTGCGGATATCAACACTCTTGATTGCAGTTTCAACGG-3′. Briefly, 2 × 10^6^ cells were seeded in 75 cm^2^ dish. Medium containing lentivirus and polybrene (8 µg/ml) was added at a multiplicity of infection (MOI) of 10 and mixed with the cells. Polybrene was used to improve infection efficiency. After incubation for 24 h, supernatants in the cells were replaced by DMEM containing FBS and cultured for 24 and 48 h for subsequent analyses.

### Statistical analysis

Statistical analysis of the data was done by using GraphPad Prism 4. Student’s *t* test was used to compare mean values between two groups. Final values are expressed as mean ± SEM. A difference of P < 0.05 was considered statistically significant.

## Results

### LPS enhanced the metastasis potential of colon cancer cells in vitro

**Fig. 1:**
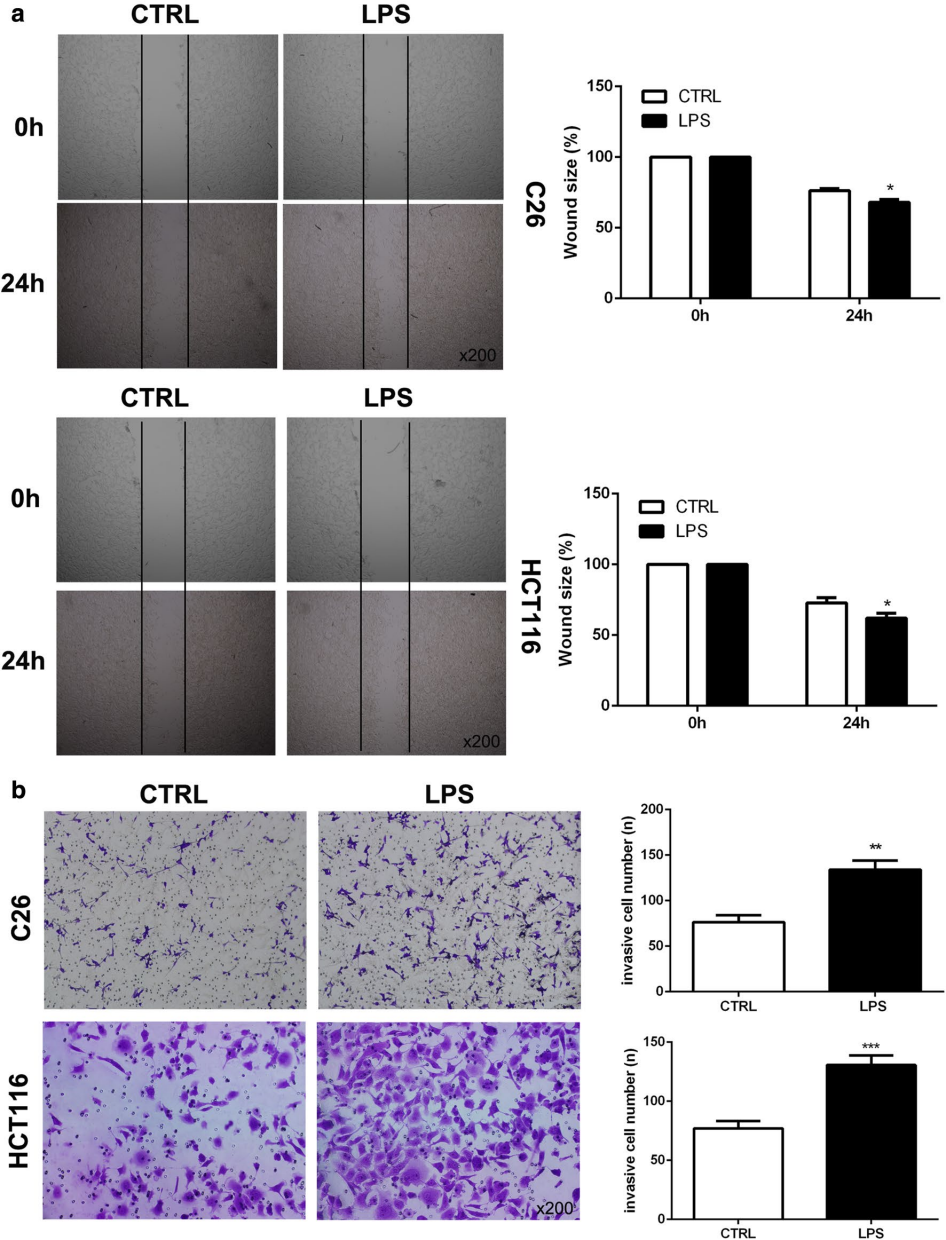
LPS enhanced the metastasis potential of colon cancer cells in vitro. LPS (10 μg/ml) was used to pretreat C26 and HCT116 cells. **a** Wound healing assay was used to examine the migration of C26 and HCT116 cells. **b** Transwell assay was employed to detect the metastasis of C26 and HCT116 cells. (*P < 0.05; **P < 0.01; ***P < 0.001)

Firstly, we employed wound healing and transwell assay to explore the role of LPS on the metastasis of C26 and HCT116 cells in vitro. As shown in Fig. 1a, b, compared with control group, LPS (10 μg/ml) could significantly promote the migration and invasion of C26 and HCT116 cells. These results indicated that LPS could enhance the colon cancer cells metastasis potential in vitro.

### Aspirin pretreatment decreased the metastasis capacity of colon cancer cells induced by LPS

**Fig. 2:**
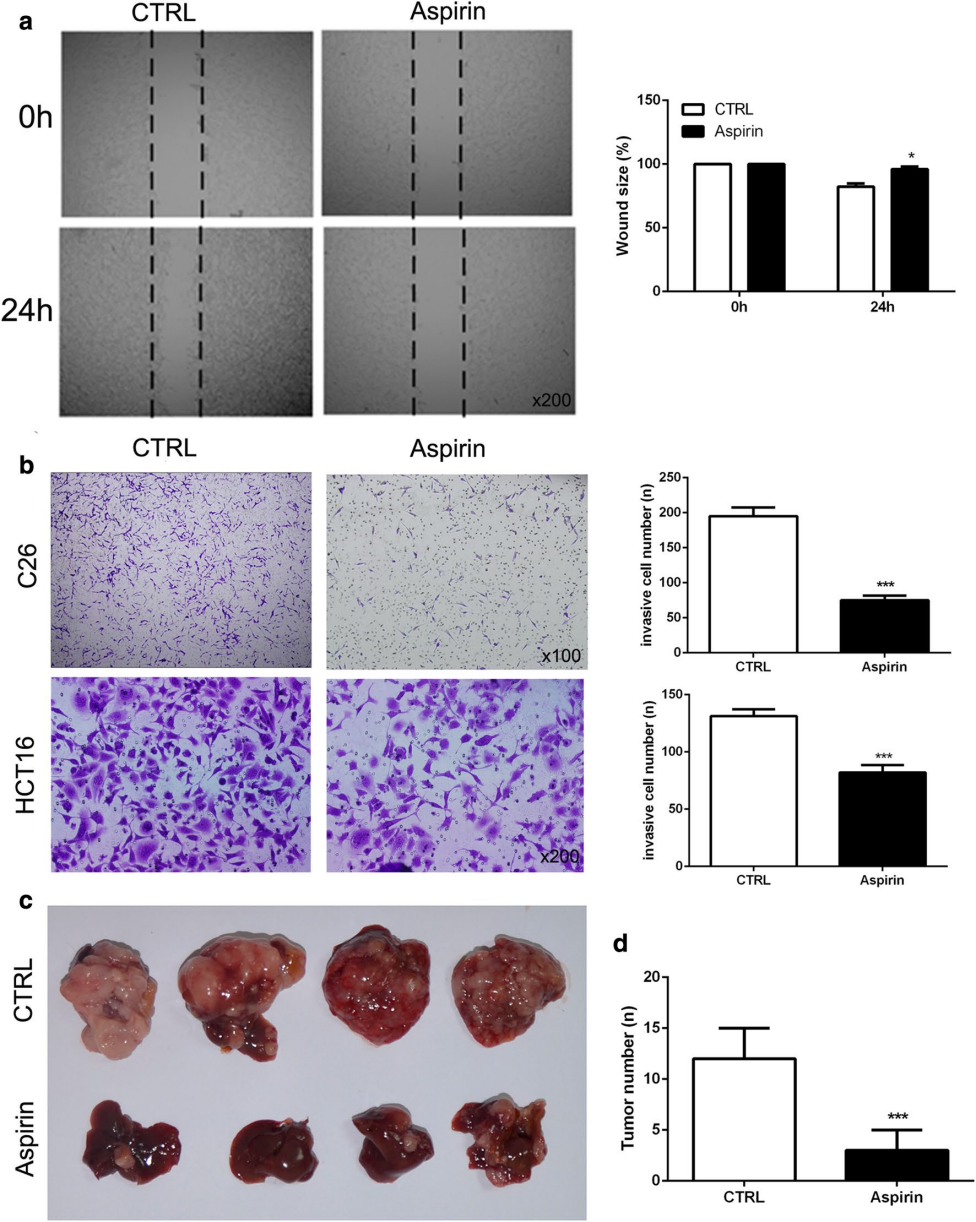
Aspirin pretreatment decreased the metastasis capacity of colon cancer cells induced by LPS. C26 and HCT116 cells were firstly exposed to aspirin (10 mmol/L) for 24 h and then LPS (10 μg/ml) was used to stimulate colon cancer cells. **a** Wound healing assay was used to examine the migration of C26 cells. **b** Transwell assay was employed to detect the metastasis of C26 and HCT116 cells. **c** Mouse splenic vein metastasis assay was used to observe the metastasis of C26 cells in vivo. **d** Tumor number was counted. (*P < 0.05; ***P < 0.001)

We have found that LPS could effectively increase the metastasis of colon cancer cells. Then cells were firstly exposed to aspirin (10 mmol/l) for 24 h hours and then wound healing and transwell assay were used to detect the effect of LPS on the metastasis of colon cancer cells. The results showed the stimulation from LPS could not lead to enhancement of the metastasis capacity in cells when they were pre-stimulated with aspirin (Fig. 2a, b). We also employed mouse splenic vein metastasis assay to examine the role of aspirin on the liver metastasis of C26 cells. As shown in Fig. 2c, d, aspirin could effectively inhibit that liver metastasis of C26 cells compared with control groups. The results indicated that aspirin could inhibit the metastasis of colon cancer cells which increased by LPS.

### LPS induced the EMT of colon cancer cells via expression of TLR4

**Fig. 3:**
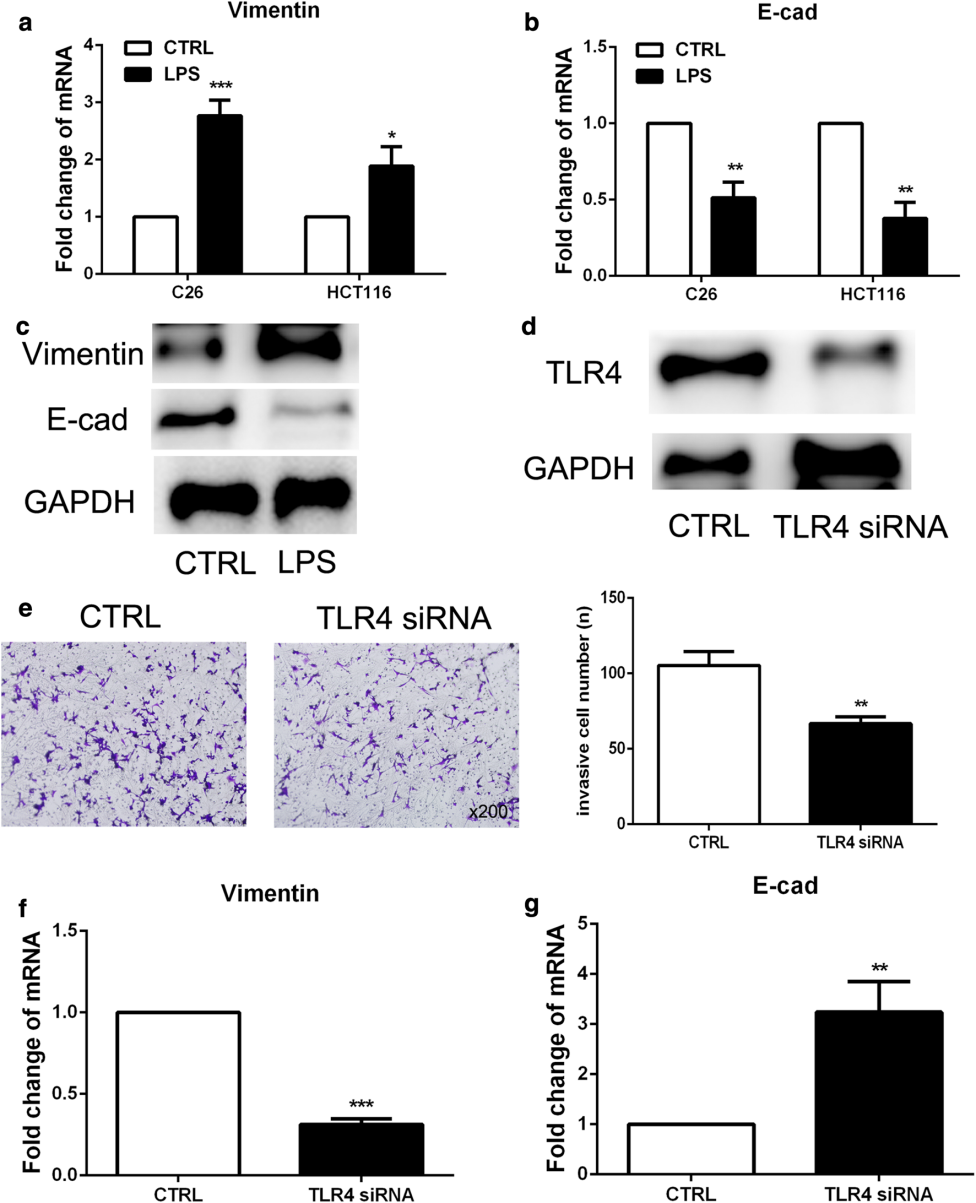
LPS induced the EMT of colon cancer cells via expression of TLR4. **a**, **b** Real-time PCR was employed to observe the effect of LPS on the EMT level of C26 and HCT116 cells. **c** Western blot was used to detect the expression of Vimentin and E-cadherin in C26 cells. **d** The inhibitory effect of siRNA on TLR4 expression in C26 cells was examined by western blot. **e** Transwell assay was used to detect the metastasis of C26 cells with TLR4 siRNA treatment. **f**, **g** Real-time PCR was employed to observe the EMT level of C26 cells when exposed to LPS with or without the expression of TLR4. (*P<0.05, **P < 0.01, ***P < 0.001)

Epithelial-mesenchymal transition (EMT) has been reported be correlated with the tumor metastasis [18]. Therefore, we use real time-PCR and immunofluorescence to observe the EMT markers in colon cancer cells which exposed to LPS. As shown in Fig. 3a, b, LPS induced the different expression of EMT markers in C26 and HCT116 cells including down-regulation of epithelial markers E-cadherin and up-regulation of mesenchymal markers Vimentin. In addition, the western blot results demonstrated that Vimentin expression was upregulated and E-cadherin was downregulated in LPS-treated colon cancer cells (Fig. 3c). These results indicated that LPS induced the EMT phenotype in colon cancer cells.

Toll-like receptor 4 (TLR4) has been reported to play important role in mediated the biological function of LPS. We examined the role of TLR4 in mediating the function of LPS. The expression of TLR4 in C26 cells was inhibited by specific siRNA (Fig. 3d) and then LPS-induced invasive ability of C26 cells was detected by transwell assay, and the results revealed that LPS-enhanced the invasion was decreased in C26 cells when TLR4 expression was silenced (Fig. 3e). In consistent,EMT phenotype was also diminished when TLR4 expression was inhibited (Fig. 3f, g). The above results demonstrated that LPS induced the metastasis and EMT phenotype of colon cancer cells via a TLR4-dependent manner.

### Aspirin decreased EMT in colon cancer cells which enhanced by LPS through decreasing the expression of TLR4

**Fig. 4:**
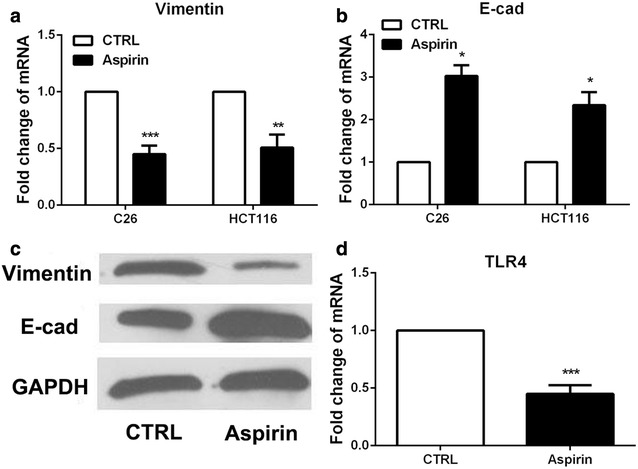
Aspirin decreased migration and EMT in colon cancer cells that enhanced by LPS by decreasing the expression of TLR4. **a**, **b** Real-time PCR was used to examine EMT markers expression in C26 and HCT116 cells with aspirin treatment. **c** Western blot was employed to detect the expression of EMT markers in C26 cells with aspirin treatment. **d** Real-time PCR was used to examine the expression of TLR4 in C26 cells with aspirin treatment. (*P < 0.05, ***P < 0.001)

EMT phenotype was also examined in aspirin-treated C26 cells and HCT116 cells. And as expected, EMT phenotype was diminished when aspirin was present (Fig. 4a–c) in C26 cells and HCT116 cells. To determine whether TLR4 was involved in the role of aspirin on the migratory phenotype which induced by LPS, we detected TLR4 expression in C26 cells after exposing to aspirin. As expected, TLR4 expression was decreased by aspirin treatment (Fig. 4d). The above data showed that aspirin decreased LPS-induced EMT by decreasing TLR4 expression.

### Inhibitory effect from aspirin on the expression of TLR4 on colon cancer cells leaded to the downregulation of NF-κB

**Fig. 5:**
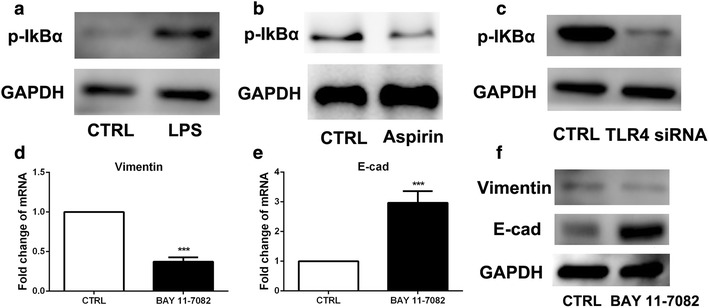
Inhibitory effect from aspirin on the expression of TLR4 on colon cancer cells leaded to the downregulation of NF-κB. **a**–**c** Western blot was used to explore the IκB activation in C26 cells with or without LPS, aspirin and TLR4 siRNA. **d**–**f** EMT markers expression was examined by real-time PCR and western blot in C26 cells with BAY 11-7082 treatment. (***P < 0.001)

Previous research has indicated that LPS could activate the nuclear factor-κB (NF-κB) signal transduction pathway and NF-κB signaling pathway was involved in the EMT process [19]. To investigate whether NF-κB pathway is required for EMT in response to aspirin, western blot was used to detect the IκB activation. The result showed that phosphorylated-IκB was activated by LPS and downregulated in C26 cells with aspirin treatment (Fig. 5a, b). In addition, TLR4 siRNA treatment clouds also inhibit the activation of IκB (Fig. 5c). Furthermore, BAY11-7082, a NF-κB inhibitor was used to block NF-κB signaling, and then LPS-induced EMT phenotype was detected. The results showed that EMT phenotype was attenuated when NF-κB signaling pathway was blocked (Fig. 5d–f). These findings indicated that aspirin inhibited migratory potential of C26 cells induced by LPS through downregulating NF-κB signaling pathway.

## Discussion

Aspirin has been reported that aspirin not only can reduce the occurrence of CRC but also reduces the risk of metastasis in CRC [9, 10]. Besides that, aspirin also could repress the development of osteosarcoma and increase the chemotherapy sensitivity of osteosarcoma by inhibiting NF-κB pathway [20]. Therefore, aspirin is concerned as candidate agency to prevent tumorigenesis. Therefore it is important to investigate the potential mechanism of aspirin inhibiting the development of tumors.

EMT is a process by which the mesenchymal phenotype is acquired by epithelial cells, which is a key process in a variety of human epithelial tumors [21]. The expression of mesenchymal markers, such as N-cadherin, vimentin, and the loss of E-cadherin, β-catenin are key molecular events of EMT. Transcription factors, such as Twist, bind to consensus E-box sequences in the E-cadherin gene promoter and down-regulate E-cadherin transcription. It is well known that EMT-like attributes contribute to the invasive phenotype and metastatic capacity of the migratory subpopulation in CRCs. This is in line with our findings that EMT phenotype is occurrence in the process of LPS induced the metastasis potential of colon cancer cells. And when the migratory features were inhibited by aspirin, EMT phenotype was also diminished.

LPS plays an important role in occurrence, development and metastasis of tumors [11–13]. In addition, LPS could also increase liver metastasis of human CRC cells [14, 15]. And the function of LPS depends on the expression of TLR4 on the cells [22]. TLR4 as a receptor of LPS has been detected in many human cancer cell lines, including pancreatic, liver and colorectal cancer [11, 14, 23]. And TLR4 plays a key role in connecting inflammation and cancer invasion and progression [24, 25]. Cammarota et al. analyzed 116 tissue samples from patients with different stages of colorectal disease and found that adenocarcinoma patients with higher TLR-4 expression in the stromal compartment had a significantly increased risk of disease progression and relapsed significantly earlier than those with lower expression levels [26]. In present study, we found that LPS induced the occurrence of EMT phenotype, which depended on the upregulation of TLR4 expression. Additionally, aspirin treatment leads to the downregulation of TLR4 on colon cancer cells which resulted in the decrease of colon cancer cells migration and EMT that induced by LPS. LPS activate NF-κB signaling pathway via binding to the receptor TLR4, which is involved in noninfectious inflammatory diseases, such as tumor invasion and survival [23]. Our work have demonstrated that activation of NF-κB signaling pathway was participated in the LPS-induced EMT and the invasive potential of colon cancer cells is in a TLR4 dependent manner and that aspirin inhibited the EMT and metastasis by down-regulating the NF-κB signaling pathway. Our data suggest that aspirin as a potential inhibitor for LPS-induced EMT and metastasis of colon cancer cells by decreasing the expression of TLR4 and NF-κB signaling.

In conclusion, we demonstrated that LPS induced metastasis ability of colon cancer cells in vitro and in vivo, and EMT phenotype was occurrence in this process. Additionally, aspirin could inhibit the migratory feature and EMT which induced by LPS. Moreover, TLR4-depended activation of NF-κB signaling pathway was involved in the promotion of migration and EMT phenotype induced by LPS. These findings indicated that aspirin might as an inhibitor in the LPS-induced metastasis of colon cancer and TLR4 could be concerned as a prognostic marker and a potential therapeutic target for LPS-induced EMT and metastasis of colon cancer.

## Abbreviations

CRC: colorectal cancer
LPS: lipopolysaccharide
NF-κB: nuclear factor kappa b
EMT: epithelial-mesenchymal transition (EMT)
TLR4: toll-like receptor 4
siRNA: small interfering RNA
